# Tools Needed to Support Same-Day Diagnosis and Treatment of Current Hepatitis C Virus Infection

**DOI:** 10.1093/infdis/jiad177

**Published:** 2023-09-22

**Authors:** Gregory P Fricker, Marc G Ghany, Jorge Mera, Benjamin A Pinsky, John W Ward, Raymond T Chung

**Affiliations:** Department of Medicine, Massachusetts General Hospital, Boston, Massachusetts, USA; Liver Diseases Branch, National Institute of Diabetes and Digestive and Kidney Diseases, National Institutes of Health, Bethesda, Maryland, USA; Infectious Diseases, Cherokee Nation Health Services, Tahlequah, Oklahoma, USA; Department of Medicine, Division of Infectious Diseases, University of New Mexico Health Sciences Center, Albuquerque, New Mexico, USA; Department of Pathology, Stanford University School of Medicine, Stanford, California, USA; Department of Medicine, Division of Infectious Diseases and Geographic Medicine, University School of Medicine, Stanford, CaliforniaUSA; Coalition for Global Hepatitis Elimination, The Task Force for Global Health, Atlanta, Georgia, USA; Department of Medicine, Massachusetts General Hospital, Boston, Massachusetts, USA; Hubert Department of Global Health, Rollins School of Public Health, Emory University, Atlanta, Georgia, USA; Hepatology and Liver Center, Massachusetts General Hospital, Boston, Massachusetts, USA

**Keywords:** diagnostic testing, hepatitis c elimination, hepatitis c virus infection, point of care testing, same-day diagnosis and treatment

## Abstract

The current multiday diagnosis and treatment paradigm for hepatitis C virus (HCV) infection results in far fewer patients receiving treatment with direct-acting antiviral agents than those with diagnosed HCV infection. To achieve HCV elimination, a paradigm shift in access to HCV treatment is needed from multiday testing and treatment algorithms to same-day diagnosis and treatment. This shift will require new tools, such as point-of-care (POC) antigen tests or nucleic acid tests for HCV and hepatitis B virus (HBV) and nucleic acid tests for human immunodeficiency virus (HIV) that do not require venous blood. This shift will also require better use of existing resources, including expanded access to HCV treatment and available POC tests, novel monitoring approaches, and removal of barriers to approval. A same-day diagnosis and treatment paradigm will substantially contribute to HCV elimination by improving HCV treatment rates and expanding access to treatment in settings where patients have brief encounters with healthcare.

Current hepatitis C virus (HCV) prevention efforts and treatment rates must improve for the United States to achieve World Health Organization global elimination targets by 2030 [1]. Within the current multiday diagnosis and treatment paradigm for HCV infection, there is a substantial loss in the cascade of care, resulting in far fewer patients receiving treatment with direct-acting antiviral agents (DAAs) than those with HCV infection diagnosed at initial testing [2, 3]. These losses are further compounded by the limited number of primary healthcare providers available to treat HCV, the difficult referral system providers have to navigate to direct patients with newly diagnosed HCV infection into care, and the lengthy time needed to procure DAAs through prior authorization requirements [4].

The American Association for the Study of Liver Diseases/Infectious Diseases Society of America guidance recommends treatment for all patients with acute or chronic HCV infection, except those with short life expectancies that cannot be remediated by HCV therapy, liver transplantation, or another directed therapy, with a goal of reducing the all-cause mortality rate and liver-related health adverse consequences through virologic cure achieved by sustained virologic response (SVR) 12 weeks after treatment discontinuation [5]. Selection and initiation of a DAA regimen can often be delayed by the time required to complete pretreatment assessments.

## CURRENT PRACTICE, DIAGNOSTIC TOOLS, AND GAPS IN CARE

Currently, pretreatment assessments start with testing to detect the presence of HCV antibody, followed, if antibody is detected, by quantitative HCV RNA testing to confirm current HCV infection. To prepare a patient for DAA therapy, clinical evaluations include testing for hepatitis B virus (HBV) and human immunodeficiency virus (HIV) infection, pregnancy status for individuals of childbearing potential, hepatic function and basic metabolic panels, noninvasive assessment to detect advanced fibrosis using clinical examination complemented by serum markers, and elastography if available [5]. Coincident HBV or HIV infection requires additional clinical management specific to these viruses. Evaluating for the presence of advanced fibrosis or cirrhosis, prior treatment failure, or HIV or HBV coinfection determines the need for specialist consultation and the need for hepatocellular carcinoma (HCC) and esophageal variceal screening after treatment [5].

While HCV eradication reduces the HCC risk dramatically, the presence of cirrhosis is associated with reduced but not eliminated rates of HCC after SVR, underscoring the need for ongoing surveillance in this population [6–11]. Aside from patients with cirrhosis infected with HCV genotype 3, in whom resistance testing may be needed before DAA selection, the presence of compensated cirrhosis or identification of genotype does not affect the duration of treatment or selection of DAA regimen now that pangenotypic regimens are available [12, 13]. Indeed, treatment of patients in low- and middle-income settings has been highly successful using a minimal monitoring approach without genotyping and dispensation of full curative courses of DAA treatment [14].

Noninvasive assessment of fibrosis and cirrhosis is straightforward and includes physical examination findings and measurement of platelet count and serum markers, and it may include elastography as well as other imaging modalities. Physical examination findings may individually have high specificity, but each has limited sensitivity and are examiner dependent [15] (Table 1). There are many direct and indirect serum markers of liver fibrosis that can aid in detecting advanced fibrosis or cirrhosis in patients with HCV infection. Some of these tests include commonly performed studies such as platelet count and aspartate aminotransferase (AST)/alanine aminotransferase (ALT), which, combined with an individual's age, can provide moderate sensitivity and specificity for the detection of fibrosis and cirrhosis (Table 2 and Table 3) [16, 17].

**Table 1.** Diagnostic Performance of Examination Findings for Cirrhosis^a^

Finding	Sensitivity (95% CI)	Specificity (95% CI)
Ascites	0.34 (.22–.49)	0.95 (.89–.98)
Collateral circulation	0.42 (.26–.61)	0.94 (.71–.99)
Encephalopathy	0.15 (.06–.33)	0.98 (.97–.99)
Firm liver	0.68 (.55–.79)	0.75 (.62–.85)
Jaundice	0.36 (.25–.48)	0.85 (.80–.89)
Spider angiomas	0.50 (.39–.61)	0.88 (.75–.95)

Abbreviation: CI, confidence interval.

^a^Adapted from de Bruyn and Graviss [15, table 3].

**Table 2.** Diagnostic Performance of Laboratory Tests for Fibrosis^a^

Test	POC Test Approved in the US for Laboratory Component	Cutoff for Specificity and Sensitivity	Sensitivity, Median (Range)	Specificity, Median (Range)	AUROC, Median (Range)	Positive Likelihood Ratio (Range)	Negative Likelihood Ratio (Range)
AST-platelet ratio index	Yes	>0.5 to >0.55	0.81 (0.29–0.98)	0.55 (0.10–0.94)	0.77 (0.58–0.95)	1.8 (1.1–4.8)	0.35 (0.08–0.78)
		>1.5 or ≥ 1.5	0.37 (0–0.72)	0.95 (0.58–1.0)	…	7.4 (1.1–15)	0.66 (0.32–1)
FIB-4	Yes	>1.45 or ≥1.45	0.64 (0.62–0.86)	0.68 (0.54–0.75)	0.74 (0.61–0.81)	2.0 (0.88–2.6)	0.53 (0.21–1.3)
		>3.25	0.50 (0.28–0.86)	0.79 (0.59–0.99)	…	2.4 (1.3–4.2)	0.63 (0.21–0.80)
FibroSURE	No	>0.10 to >0.22	0.92 (0.88–0.97)	0.38 (0.27–0.56)	0.79 (0.70–0.89)	1.5 (1.3–1.9)	0.21 (0.11–0.28)
		>0.70 or >0.80	0.22 (0.20–0.50)	0.96 (0.95–0.98)	…	5.5 (5.5–13)	0.81 (0.53–0.82)
ELF test	No	>8.75, >9.0, or >9.78	0.85 (0.84–0.86)	0.70 (0.62–0.80)	0.81 (0.72–0.87)	2.8 (2.3–4.2)	0.21 (0.19–0.23)
FIBROSpect II	No	>0.36 or ≥0.42	0.80 (0.67–0.95)	0.70 (0.66–0.74)	0.86 (0.77–0.90)	2.6 (2.4–2.9)	0.29 (0.08–0.45)

The two rows included for AST-platelet ratio index, FIB-4, and FibroSURE represent diagnostic performance of these tests at different cutoff values. Abbreviations: AST, aspartate aminotransferase; AUROC, area under the receiver operating characteristic curve; ELF, enhanced liver fibrosis; FIB-4, Fibrosis-4 index; POC, point-of-care.

^a^Adapted from Chou and Wasson [16, table 2]. Fibrosis was defined as METAVIR stages F2–F4, Ishak stages 3–6, or the equivalent.

**Table 3.** Summary of Diagnostic Performance of Laboratory Tests for Cirrhosis^a^

POC Test	Approved in the US for Laboratory Component	Cutoff for Specificity and Sensitivity	Sensitivity, Median (Range)	Specificity, Median (Range)	AUROC, Median (Range)	Positive Likelihood Ratio (Range)	Negative Likelihood Ratio (Range)
AST-platelet ratio index	Yes	>1.0 or ≥1.0	0.77 (0.33–1.0)	0.75 (0.30–0.87)	0.84 (0.54–0.97)	3.1 (1.4–4.9)	0.31 (0–0.77)
		>2.0 or ≥2.0	0.48 (0.17–0.76)	0.94 (0.65–0.99)	…	8.0 (1.4–18)	0.55 (0.27–0.84)
FIB-4	Yes	>1.45	0.90	0.58	0.87 (0.83–0.92)	2.1	0.17
		>3.25	0.55	0.92	…	6.9	0.49
FibroSURE	No	>0.56 or >0.66	0.85 and 0.82	0.74 and 0.77	0.86 (0.71–0.92)	3.3 and 36	0.20 and 0.23
		>0.73, >0.75, or >0.8962	0.56 (0.30–1.0)	0.81 (0.24–0.96)	…	2.9 (1.2–10)	0.54 (0–0.79)
ELF test	No	Varied	Not calculated	Not calculated	0.88 (0.78–0.91)	Not calculated	Not calculated

The two rows included for AST-platelet ratio index, FIB-4, and FibroSURE represent diagnostic performance of these tests at different cutoff values. Abbreviations: AST, aspartate aminotransferase; AUROC, area under the receiver operating characteristic curve; ELF, enhanced liver fibrosis; FIB-4, Fibrosis-4 index; POC, point-of-care; US, United States.

^a^Adapted from Chou and Wasson [16, table 3]. Cirrhosis was defined as METAVIR stage F4, Ishak stages 3–6, or the equivalent.

Other tests used to detect fibrosis are proprietary indices that perform well for the detection of fibrosis and cirrhosis, such as FibroSURE ,which measures indirect markers of fibrosis, and the enhanced liver fibrosis test and FIBROSpect II, which are direct measurements of extracellular matrix turnover [16] (Tables 2 and 3). These are valuable tests to aid in the diagnosis of advanced fibrosis, but while point-of-care (POC) tests for AST, ALT, and complete blood cell count are available, there are unfortunately no such tests for the proprietary indices, which require central laboratory processing. As an alternative, the use of other calculated indices that rely on widely available peripheral blood tests—such as the Fibrosis-4 index, which incorporates AST, ALT, platelet count, and patient age, or the AST-platelet ratio index, which incorporates platelet count, AST, and AST upper limit of normal—may be an efficient means of staging patients at the POC [18].

Imaging evaluation of advanced fibrosis can be performed with ultrasonography (US), using transient or shear-wave elastography to assess liver stiffness (with sensitivities of 0.78 and 0.74 and specificities of 0.87 and 0.84, respectively, for the detection of advanced fibrosis) with the measurement of an induced shear wave or acoustic radiation force impulse to measure the speed of an acoustically generated shear wave with comparable diagnostic performance [19–22]. Magnetic resonance (MR) elastography is less operator dependent than US-based methods, with fewer technical failures (area under the receiver operating characteristic curve for MR elastographic detection of ≥F3 fibrosis, 0.94 [95% confidence interval, .91–.95]) [23–26]. However, access to both transient and MR elastography is limited. Standard imaging modalities, including abdominal US, computed tomography, and MR imaging, may identify stigmata of cirrhosis, such as liver nodularity, splenomegaly, and varices, but they have limited sensitivity. Each of these modalities have restricted accessibility at most points of care.

Currently, tests to diagnose current HCV infection must be performed in the clinical laboratory, which may result in delays to treatment and is a recognized barrier in the care cascade. The Clinical Laboratory Improvement Amendment (CLIA) program regulates clinical laboratories that perform tests approved and cleared by the Food and Drug Administration (FDA), including both waived and nonwaived tests, as well as laboratory-developed tests. CLIA-waived tests are simple, approved tests with low risk for an incorrect result and fewer regulatory requirements [27]. POC tests include both waived and nonwaived tests. There are no FDA-approved, CLIA-waived tests to detect current HCV infection. HCV nucleic acid tests (NATs) and core antigen (cAg) tests require venous blood and processing in a central laboratory to make a diagnosis of HCV infection. There is a single FDA-approved, CLIA-waived anti-HCV antibody test, the Oraquick HCV rapid antibody test. There are other anti-HCV antibody tests that are suitable for the POC and prequalified by World Health Organization and could be submitted to FDA for licensure and use in the United States [18]. However, HCV antibody testing alone cannot distinguish between acute infection, cleared infection, chronic infection, reinfection, and infection in immunocompromised patients [28]. In addition, there are no FDA-approved, CLIA-waived HBV NAT or hepatitis B surface antigen (HBsAg) tests. Only HIV antibody and antigen-antibody combination tests are readily available for use at the POC in the United States and globally [29].

Successful national HCV elimination programs in Egypt, Georgia, and Rwanda have combined anti-HCV POC testing with reflex laboratory-based RNA testing with high rates of linkage to care, DAA treatment uptake, and achievement of SVR [30–33]. In the United States, about 40% of people with chronic HCV infection are unaware of their infection [34]. Also in the United States, as few as 52% of patients with positive anti-HCV antibody underwent HCV RNA testing, and of those with positive RNA results, only 65% were engaged in care and 22% were prescribed DAA therapy [35]. The estimated pooled DAA treatment uptake among patients with diagnosed HCV infection and positive HCV RNA results in the United States was a mere 29% (95% confidence interval, 18%–40%) [36]. In addition to adherence to screening guidelines and improving transition through the HCV treatment cascade, DAA uptake requires diagnosis to be paired with treatment. Many states continue to impose restrictions on the prescription of DAAs, including requirements for prior authorization, fibrosis testing, sobriety, and specialist prescribers [37]. The success of a novel minimal monitoring approach to HCV treatment and the dispensing of a complete DAA course without such restrictions offers a framework for practical implementation of simplified treatment programs [14].

To follow these recommendations and meet the needs of patients with HCV infection, new tools and a new diagnostic and treatment algorithm are needed for same-day diagnosis and treatment. Performing pretreatment assessments in a same-day treatment paradigm will require rapid, accurate, and affordable testing options, rapid risk stratification and connection to care, and changes in payer policies in the United States regarding DAA approval.

## STEPS TOWARD SAME-DAY TESTING AND CURE

To identify patients with acute or chronic HCV infection, through the recommended 1-time testing of adults in the United States, inexpensive, rapid, and reliable FDA-approved, CLIA-waived POC tests (HCV NAT or HCV cAg that can be performed on a capillary blood or oral swab sample) are needed to complement anti-HCV antibody tests. A consensus-based target product profile for such a POC test of HCV viremia outlined a minimum required sensitivity of 90% [38]. A large multinational study of patients with HCV viremia described limits of detection of 3311, 1318, and 214 IU/mL to achieve sensitivities of 95%, 97%, and 99%, respectively, for the detection of HCV viremia [39]. While there is not an FDA-approved HCV cAg test in the United States, HCV cAg has been shown to be an effective and sensitive screening test for detecting HCV infection. For example the Abbott HCV cAg test and Roche Elecsys Duo HCV cAg-antibody combination assays are available in Europe [40–46], though these tests require laboratory infrastructure and cannot be performed at the POC.

Once a diagnosis of HCV infection has been established, patients can be counseled on the risks and benefits of treatment. POC pregnancy testing is recommended for individuals of childbearing potential. If results are positive, HCV treatment can be considered during pregnancy on an individual basis after a patient-physician discussion about the potential risks and benefits, recognizing that there are no DAAs with FDA approval for use in pregnancy [47–49]. Patients with a history or clinical features of cirrhosis and coinfection with HIV or HBV and those who are DAA treatment experienced may require subspecialty consultation. Most patients with HCV infection could start treatment the day of diagnosis. To achieve this, rapid, accurate, and affordable FDA-approved, CLIA-waived POC HBV NAT or HBsAg tests are needed that can be performed on capillary blood or oral swab samples. While laboratory-based HBV NAT and HBsAg tests are commonplace, there are no CLIA-waived POC tests available. It is hoped that this will change soon, as screening for HBV is also essential to the goal of HBV elimination laid out by the World Health Organization. The combination of HCV and HBV antigen testing or NAT with HIV antibody-antigen testing or NAT in a single multiplex POC assay could further simplify testing for these cotransmitted blood-borne pathogens. Currently, there are multiplex NAT assays for HIV, HBV, and HCV that have been FDA approved for use in screening before blood and organ donation but are not cleared for disease screening [50].

To further determine who should be referred for additional care after initiating treatment on the day of diagnosis, patients could be assessed for advanced fibrosis based on the Fibrosis-4 index and/or the AST-platelet ratio index by using available CLIA-waived tests for AST, ALT, and complete blood cell count. Other assays, such as FibroSURE, the enhanced liver fibrosis test, or FibroSPECT II, may help identify those requiring future subspecialty consultation and HCC and variceal screening. Similarly, increased access to elastography would support a same-day treatment paradigm. With increased linkage to care, more patients may require subspecialty referral, and access to subspecialists when needed for HCV-related care should be increased. Given bottlenecks in access to subspecialty care and projections for shortfalls in the hepatology workforce [51], education of and task sharing by generalists, primary care providers, and community health workers will be essential for managing the long-term care of newly identified persons with HCV infection.

Just as important, same-day treatment would require removal of barriers to approval of DAAs by payers. Requirements by insurers for genotyping, fibrosis staging, patient sobriety before approval or reimbursement of HCV prescriptions, and preapproval authorization are not in patients’ interest and must be removed in light of strong evidence for high cure rates across all patient populations receiving DAAs [5, 52–55]. To accomplish this, regimens need to be preapproved and DAAs available on site so treatment can begin on the day of diagnosis at the POC.

Looking to the future, with the availability of POC testing, the development of an effective long-acting injectable DAA could also substantially improve treatment rates in patients with HCV infection who have little contact with the traditional healthcare system and could be a valuable tool for HCV elimination. Long-acting injectable HIV therapy has demonstrated noninferiority to daily dosing with dosing intervals as long as every 2 months [56–59]. While viral suppression requires ongoing therapy for HIV treatment, high rates of SVR are achieved with DAAs for HCV infection. The potential for a similar long-acting injectable DAA for HCV infection to induce SVR with shorter or even single-dose regimens could open access to same-day treatment and even SVR in difficult-to-access patient populations.

**Figure 1.**
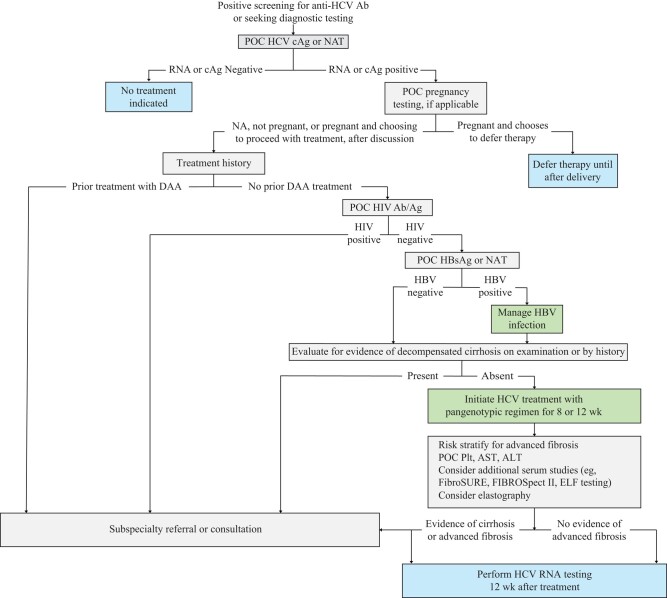
Same-day diagnosis and treatment algorithm. At the time of publication, no hepatitis C virus (HCV) core antigen (cAg) assay has been approved in the United States. HCV treatment can be considered during pregnancy on an individual basis after a patient-physician discussion about the potential risks and benefits. Among patients with HCV infection, up to 5% have coinfection with human immunodeficiency virus (HIV), and up to 15% have coinfection with hepatitis B virus (HBV) [60, 61]. Patients with HIV infection should start antiretroviral therapy before starting treatment with a direct-acting antiretroviral agent (DAA). Patients with HBV infection should receive appropriate treatment and may require subspecialty consultation. The combination of HCV and HBV antigen (Ag) or nucleic acid testing (NAT) and HIV antibody (Ab)/Ag or NAT into a single multiplex point-of-care (POC) assay could further simplify testing for these cotransmitted blood-borne pathogens. Evidence of cirrhosis or advanced fibrosis should prompt referral for management and screening. Abbreviations: ALT, alanine aminotransferase; AST, aspartate aminotransferase; ELF, enhanced liver fibrosis; NA, not applicable; Plt, platelet count.

To achieve HCV elimination, a paradigm shift in access to HCV treatment is needed, from current multiday testing and treatment algorithms to same-day diagnosis and treatment (Figure 1). This shift will require new tools, such as FDA-approved, CLIA-waived POC antigen or NAT for HCV and HBV and NAT for HIV that do not require venous blood and better use of existing resources, expanding HCV screening and treatment by primary care providers, with improved access to HCV treatment through the availability of on-site treatment, removal of payer barriers to approval, adoption of minimal monitoring approaches during treatment, expanded access to available POC tests, and available specialist referral networks for patients who fail initial therapy, have cirrhosis, or have coincident HIV or HBV infection. A same-day diagnosis and treatment paradigm will substantially contribute to HCV elimination by improving treatment rates for those with diagnosed HCV infection and expanding access to treatment in settings where patients have brief encounters with healthcare providers: substance use disorder treatment facilities, syringe service programs, mobile treatment programs, correctional facilities, federally qualified health clinics, inpatient wards, and emergency departments.

## Notes

***Financial support*.** This work is supported by the National Institute of Allergy and Infectious Diseases (grant R25 AI147393 to G. P. F.)

***Supplement sponsorship.*** This article appears as part of the supplement “Towards a Single-Step Diagnosis of Hepatitis C Virus Infection,” sponsored by the Centers for Disease Control and Prevention.
